# ‘Back to better’: amplifying health equity, and determinants of health perspectives during the COVID-19 pandemic

**DOI:** 10.1177/17579759211000975

**Published:** 2021-03-24

**Authors:** Sume Ndumbe-Eyoh, Pemma Muzumdar, Claire Betker, Diane Oickle

**Affiliations:** National Collaborating Centre for Determinants of Health, Antigonish, Nova Scotia, Canada

**Keywords:** determinants of health, equity / social justice, systems, policy / politics, community action

## Abstract

**Introduction:**

Equity and social justice have long been key tenets of health promotion practice, policy and research. Health promotion foregrounds the pertinence of social, economic, cultural, political and spiritual life in creating and maintaining health. This necessitates a critical structural determinants of health perspective that actively engages with the experiences of health and wellbeing among diverse peoples. The inequitable impacts of pandemics are well documented, as are calls for improved pandemic responses. Yet, current pandemic and emergency preparedness plans do not adequately account for the social and structural determinants of health and health equity.

**Methods:**

Through five one-hour online conversations held in April 2020, we engaged 13 practice, policy, research and community leaders on the intersections of COVID-19 and gender, racism, homelessness, Indigenous health and knowledge, household food insecurity, disability, ethics and equitable futures post-COVID-19. We conducted a thematic analysis of speaker and participant contributions to investigate the impacts and influence of COVID-19 related to the structural and social determinants of health. We analyzed which policies, practices and responses amplified or undermined equity and social justice and identified opportunities for improved action.

**Findings:**

Analysis of the COVID-19 pandemic revealed four broad themes:

• oppressive, unjust systems and existing health and social inequities;

• health and social systems under duress and non-responsive to equity;

• disproportionate impacts of COVID-19 driven by underlying structural and socioeconomic inequity; and

• enhanced momentum for collective mobilization, policy innovations and social transformation.

**Discussion:**

There was a strong desire for a more just and equitable society in a post-COVID-19 world, going ‘back to better’ rather than ‘back to normal.’ Our analysis demonstrates that equity has not been well integrated into pandemic planning and responses. Social movement and systems theories provide insight on ways to build on existing community mobilization and policy openings for sustained social transformation.

## Introduction

Structural determinants of health are the economic, cultural, political and social structures that shape the distribution of material and symbolic power and resources. In tandem with concerns for the health of the planet and ongoing legacies of colonialism and racism, these ‘structural drivers’ shape public policies across sectors creating predictable inequities in health and health-promoting resources across and between nations and communities (1–5). The social gradient in health illustrates that those with more relative advantage experience better health than those with less advantage.

During the 2009 influenza pandemic in England, age and sex-standardized mortality was three times higher for those in the most socioeconomically deprived areas (6). First Nations, Inuit and Métis peoples in Canada were disproportionately affected by both waves of the 2009 H1N1 pandemic (7). For example, First Nations, Inuit and Métis peoples represented 27.8% of hospital admissions during the first wave even though they made up about 4.3% of the Canadian population (7). In the United States, H1N1 led to 4 to 4.5 times higher hospitalization rates for Hispanics, Blacks and Asian/Pacific Islanders (8) than for Whites, and higher mortality for American Indians/Alaska Natives compared with other racial/ethnic groups (9). Others have documented similar disproportionate impacts of pandemics by gender (10) and disability (11).

Core health promotion values of equity and social justice foreground the pertinence of social, economic, cultural, political and spiritual life for health (12). These relationships and values are relevant to pandemics (13–15). What’s more, the inequitable impacts of pandemics are well documented, as are calls for improved responses (16–19). Yet, these core values are not integrated into current pandemic and emergency preparedness plans or responses, evidenced in the lack of intentional consideration for the social and structural determinants of health and health equity. In the Canadian context, equity is seldom named in pandemic preparedness plans (20) and the initial description of who was considered ‘vulnerable’ in the early days of the COVID-19 pandemic failed to engage equity and structural determinants of health perspectives (21).

With a goal to rapidly identify, generate and share equity-focused impacts and responses to the COVID-19 pandemic, we organized a series of online conversations for the public health community and others. We sought to answer the following questions: As the COVID-19 pandemic took hold across the globe, was the intersecting relationship between social determinants of health embodied in public health and policy responses? What were the emerging impacts of COVID-19 on social and health inequities?

## Methods

Five one-hour online facilitated conversations engaged 13 practice, policy, research and community leaders including: Indigenous elders and knowledge keepers, policy and decision makers, practitioners, and researchers from health and non-profit sectors. Speakers explored the manifestations and experiences of COVID-19 at the intersections of multiple structural and social determinants of health. Over 1600 participants registered for the series, representing disciplines and roles across public health as well as other sectors. Participants were largely from Canada with a small proportion from other countries.

Conversation themes were: 1) Health equity, determinants of health, and COVID-19, 2) Community impact and responses to COVID-19 (focus on gender, racism, homelessness), 3) Indigenous perspectives on COVID-19, 4) Community impacts and responses (focus on household food insecurity, disability and ethics), and 5) Equitable futures in a post-COVID-19 world (22). Participants contributed questions, lived experience and resources during the conversations and as part of session registration. The conversations were recorded and are posted online for public access.

We conducted a thematic analysis (23) of the content explored during the conversations guided by three research questions:

- What is the impact of COVID-19 on health equity and the social and structural determinants of health?
- Which policies, practices and responses amplify or undermine equity and social justice?
- What are the opportunities for improved action?

We adapted the six phases presented by Braun and Clarke (23) and refined by Nowell *et al*. (24) for our analysis. First, all authors attended the conversations and/or listened to the recordings, taking field notes on what themes initially emerged during the dialogues. We transcribed the recorded conversations and collated participants’ contributions after each session. We then coded data from the five conversations using tags developed from initial themes (e.g. ‘community mobilization,’ ‘collaboration’). We mapped data in tables, identifying relevant quotes from speakers and participants. Mid-way in the process, we presented a preliminary set of themes to a group of public health leaders with recent experience with the COVID-19 pandemic for validation and augmentation, and incorporated feedback in the next phase of distilling, scoping and theming. We used illustrative quotes to enrich the findings.

Speakers verbally consented to the recording and sharing of the conversation recordings. The information from the conversations is publicly available online. For that reason, we did not seek research ethics approval. Direct quotes have been anonymized. However, given that this information is in the public domain we cannot guarantee confidentiality or anonymity (25).

## Findings

Analysis of the COVID-19 pandemic revealed four broad themes:

1) oppressive, unjust systems and existing health and social inequities;
2) health and social systems under duress and non-responsive to equity;
3) disproportionate impacts of COVID-19 driven by underlying structural and socioeconomic inequity; and
4) enhanced momentum for collective mobilization, policy innovations and social transformation.

### 1) Oppressive, unjust systems and existing health and social inequities

Speakers and participants situated the pandemic as a multi-purpose device: part flashlight, part hammer, and part crystal ball.

#### a. COVID-19 as a flashlight, that illuminates existing inequities

The pandemic shone light on intersecting structures and systems that drive inequitable health and social outcomes. The direct and indirect impacts of COVID-19 made visible structural drivers of inequity, including colonization, white supremacy, racism, patriarchy, capitalism and a frail social safety net (e.g. health, employment insurance, welfare):

*COVID-19 is an example of that sort of a biophysical phenomenon and social phenomenon that’s filtered through systems in which some of our lives are deemed more essential or more valuable than others.* [S1]

More specifically, COVID-19 made systemic inequities more visible to those who experience social and economic privilege.

*What we are seeing is an emergence into the consciousness and space of the non-poor, or the non-marginalized, of some of the issues that many of us had been working on for a very long time.* [S2]

This heightened awareness of inequities within the public consciousness presented an opportunity for public health to engage with intersectoral partners and community groups, and advocate for shifts in social and economic policy.

*One of the things that COVID has done, moving forward, is that we are conscious now of some of these realities and how we have been impacted by it ..., and that’s painful, and that can be very challenging, but our responsibility in public health is to sustain consciousness.* [S14]

#### b. COVID-19 as a hammer, that applies further stress and intensifies inequities

Participants and speakers depicted the pandemic as a hammer, dealing blows to existing points of stress and intensifying inequities.

*[COVID-19] is reinforcing the existing structural inequities that are deepened in a pandemic ... hitting marginalized and disadvantaged groups the hardest. It’s like there is two COVID-19 realities. There’s those who can’t quarantine, and they are serving the rest of us who can afford to quarantine, and that’s called quarantine privilege.* [S13]

They described avoidable and unfair systems that led to even worse outcomes for those already experiencing marginalization. Outcomes included increased susceptibility to COVID-19 infection, income inequity, food and housing insecurity, and challenges for people who use substances.

*Indigenous peoples statistically have high rates of respiratory and heart disease, due to a variety of longstanding determinants of health stemming from continued colonization. These conditions make a person especially vulnerable to COVID-19.* [P1]

#### c. COVID-19 as a crystal ball for future planetary health disruptions

Speakers framed inequities caused and exacerbated by COVID-19 as a cautionary crystal ball, indicative of what would happen during disruptions due to massive ecological change. They drew connections between the emergence of COVID-19, the state of planetary relationships, and the need for justice, solidarity, and a sustainable future:

*And I think this situation is a test of our global solidarity. And we need it, because it’s from a global health or global justice perspective. And what we’re in right now is also a dress rehearsal for climate change.* [S11]

Calling for intersectional approaches and drawing links to past movements, a speaker noted that addressing racism and sexism is essential to create a sustainable future:

*Sustainable for whom is my first question ... it’s like the women’s movement, you know. Quickly we realize, okay, ... ‘Woman of colour’ is really not included in this so-called women’s movement... . So there’s a core piece around equity. There’s anti-Black racism, Indigenous racism ... [if] we don’t really articulate those and make sure they’re included in all this other work, we end up with improvement and widening inequities.* [S12]

### 2) Health and social systems under duress and non-responsive to equity

COVID-19 responses exposed that health and social systems were under duress, ill-prepared:

*... the old system never prepared us for a global pandemic. We knew the old system was reinforcing inequities and leaving people behind. And we also knew that there was a better way forward. We are seeing how the old social safety net was too frayed to really rise to the occasion.* [S13]

*The time to think about ethics in an emergency situation is before the situation itself. And when we’re talking about equity, equity has to be built into things beforehand and not on the fly.* [S11]

The erosion of public health and social systems limited the extent to which core public health functions could be fulfilled. Redeployment of existing public health resources to the COVID-19 response resulted in little or no resources left to attend to significant public health issues and health equity issues.

There was not a strong equity focus in planning, and responses did not apply past learnings about who was most likely to be impacted:

*Look at who is catching the virus, who is dying, who got the training and the protective gear and when. Long-term care, group homes, congregate living for people with mental and physical disabilities and the staff that provided care came after. We should focus on where we know the spread will occur. There isn’t anybody in public health, in regional planning, that didn’t know after SARS and H1N1 that care settings were our priorities, and yet they did not get priority.* [S12]

Measures taken did not account for those experiencing structural disadvantage. For example, physical distancing guidelines were difficult to apply in the shelter system.

The closure of supportive services and programs (e.g. daycares, schools, libraries, public spaces, businesses) significantly impacted structurally disadvantaged communities, who rely on these services for various reasons (e.g. access to food, shelter, digital connections).

Participants and speakers noted that the pandemic increased the visibility of both public health and the public’s health, and identified opportunities to improve weaknesses in health and social systems:

- Address racism, colonialism, and unfair economic structures as part of emergency preparedness planning, response and recovery.
- Develop and implement public policies, guidelines and frameworks that consider the health and social impacts of pandemics.
- Engage in proportionate universalism to account for health outcomes along a social gradient.
- Before, during, and after the emergency, strategize with community members and intersectoral providers as to how to mitigate impacts for populations experiencing social injustice (e.g. those who experience homelessness, low income, food insecurity etc).
- Apply ethical principles such as transparency and accountability when engaging with communities and intersectoral partners.

### 3) Disproportionate impacts of COVID-19 driven by underlying structural and socioeconomic inequity

COVID-19, and public health and policy responses to the virus, disproportionately impacted multiple communities. Due to the social and structural determinants of health, communities experienced specific and unequal impacts, described below.

#### a. Indigenous peoples

Jurisdictional conflicts, inadequate housing, and lack of access to clean water, all rooted in colonization, impacted the experience of COVID-19 for Indigenous peoples. Food access and supply chain issues were present for remote Indigenous communities. In some settings there were barriers to following public health measures, and guidance was not tailored for Indigenous communities. Additionally, resources for Indigenous people in hospitals were diverted to COVID-19 testing, leading to compromised care.

#### b. Black and racialized communities

Systemic racism manifested in inequities in the social determinants of health like housing, education, income, employment and health care access. The health system did not account for the impact of systemic racism on COVID-19 infections, treatment and mortality, and there was a lack of race-based data. Black communities were not referred to testing, denied care and had their symptoms minimized. Black and racialized people were over-represented in jobs deemed as essential, putting them at increased exposure to COVID-19, and did not always have access to technology to access information. Overt acts of racism and stigma, often directed to members of the Chinese community, increased interpersonal violence. Black and Indigenous communities experienced state-sanctioned violence, due to the disproportionate enforcement of public health guidelines.

#### c. Gender

Women and gender diverse people had increased exposure to COVID-19 due to overrepresentation in health, social and service sector jobs deemed as essential. Exposure was heightened for racialized women, including newcomers over-represented in low-income, temporary positions without the benefits necessary to protect workers. COVID-19 increased gender-based violence, with intimate partner violence as a hidden crisis within the pandemic for women isolating at home. As crowded shelters were unable to provide support, women were exposed to further harm.

#### d. Precariously employed

People in precarious employment, and those who were unable to work at home, experienced job and income loss. Precariously employed workers were more likely to have challenges maintaining physical distancing in their work. Many were unable to access benefits or relief supports. Women and racialized people were over-represented in part-time and essential positions without paid sick leave and less likely to have resources to manage the financial and emotion burden of working from home, performing childcare duties, and self-isolating.

#### e. Food insecure

People on social assistance and in low-wage precarious jobs experienced household food insecurity. Due to limited internet and/or or credit access, low-income communities could not always engage with the virtual solutions created to comply with public health measures (e.g. ordering food online, contactless payment, etc).

#### f. Housing insecure

People experiencing homelessness faced increased exposure to COVID-19 in shelters. Housing inadequacy worsened as shelters closed.

#### g. People with disabilities

Young women and girls with disabilities experienced greater COVID-19 exposure risk, lack of access to basic necessities and benefits, increased vulnerability in institutional care settings, uncertain availability of supports, lack of personal protective equipment and screening, and slowed or discontinued home care. Physical distancing resulted in heightened feelings of invisibility, isolation, exclusion, and lack of importance to government and society. Information was not adapted for people with intellectual and physical disabilities. People with disabilities experienced stigma and discrimination in public and professional settings through overt comments, the de-prioritization of resources, loss of income, and ableist approaches to triage for health system resources like intensive care unit beds and ventilators.

#### h. Mental health

COVID-19 significantly impacted the mental health of practitioners, essential workers and community members including anxiety, stress and community trauma.

### 4) Enhanced momentum for collective mobilization, policy innovations and social transformation

#### a. Community mobilization and organizing

Community responses rapidly emerged to meet the distinct needs of intersectional communities even as they were being deeply impacted by COVID-19. Community-based organizations were well positioned to provide services and supports, credited to the close relationships with communities, governance by community members, and strong networks:

*So these frontline organizations are already community governed, they’re run by and for communities, and so that means we can tap into those networks of staff, of peer workers, of board members, of volunteers in the community and clients, to continue to give advice through the networking structures that we already have, to help make sure that we’re making decisions that are reflective of what the community needs and wants, and what their current circumstances are.* [S2]

‘Care mongering’ at the community-level played a critical role to fill gaps left by fractured health and social systems. These self-organized schemes like mutual aid groups were largely led by equity-seeking communities who provided their communities with basic necessities. Indigenous-specific responses emerged as communities protected elders by restricting entry into their Nations. Programs like virtual traditional dancing were delivered to counteract isolation stemming from physical distancing and to maintain cultural practices. Traditional food systems, medicines and practices enhanced community wellbeing.

#### b. Policy innovations on the social determinants of health

The COVID-19 pandemic opened an unprecedented opportunity for policy change and innovations. These innovations represented years of on-the-ground research, advocacy and activities to shift policy. The speed at which policies were implemented actively challenged notions of change as incremental, an approach often implored in policy circles. Faced with the urgency of the pandemic and the hypervisibility of harm and inequities, governments moved more expediently than previously seen:

*I’ve been talking to a number of public policy advocates who have said that the amount of changes we’ve seen in public policy in the last 20 days has surpassed the changes that many people have seen in the last 20 years ...* [S5]

Sector-specific policies responded to the direct and indirect impacts of COVID-19. Early policy innovations were already being implemented in April 2020 to improve the social determinants of health by different levels of government. At the federal level, for example, the Canada Emergency Response Benefit was implemented to replace lost income due to COVID-19 (26). At the municipal level, innovations in housing were observed whereby hotels were used to provide safe housing for people experiencing homelessness. Coupled with the provision of food, medical aid, mental health supports and a drug supply, this showed positive benefits:

*... folks having their own safe space ... their own bathroom ... their own lock on the door ... we’re seeing many of the folks who are being placed into these hotels and motels having quite good outcomes ... improvement on health ... positive improvement on social outcomes as well.* [S7]

Policy approaches, such as increased funding to food banks as a charity-based solution to food insecurity and Canada Emergency Response Benefit (CERB) were critiqued for failing to address and restructure power relations, leading to calls for social transformation that went beyond sector-specific reforms.

#### c. Igniting social transformation for health and social equity

Social transformation was articulated in spiritual, political, social, economic and cultural terms. Drawing on Indigenous spirituality and knowledge, one speaker stated:

*I was always told of the Seventh Fire, the Seventh Fire of people awakening themselves, and we’re in it, we’re in the midst of the Seventh Fire. And I really thought to myself that my children’s children wouldn’t have to deal with this, right. But now it’s staring us in the face so it’s kinda woken me up, right.* [S8]

Accordingly, transformation was inevitable given the existing policy windows and the ‘enormous ... abyss’ between pre-pandemic social safety nets and the systems required to promote good health and a good life:

*I think in terms of this idea of coming back to status, quo, we can’t. CERB, as it’s set up right now, is actually way more money than those who rely on social assistance and disability benefits, so that speaks to the enormous gap, abyss, whatever you want to call it that is very ableist in how we determine people’s worth and the lives that they live. So going back to status quo is not an option.* [S9]

#### d. Power reasserts itself

Despite the potential for policy innovations and social transformation, there was skepticism of realizing a ‘better normal.’ This was grounded in the knowledge that social transformation is difficult, lack of trust in governments based on historical experiences and the contention that power seeks to reassert itself.

*I think because this is a matter of privilege and power, I do believe that the urge is going to be to restore that power, and so institutions and individuals, communities with privilege, are going to want to protect their privilege, and institutions are going to cater to that.* [S14]

Antidotes to a return to the status quo surfaced such as courage, persistent organizing, engaging those with influence, productive conflict and accountability:

*... working together to find leaders to make these move right now I think would be really helpful. Identifying what’s working and what isn’t working. And trying to identify and make the most of windows that are open or the doors that are open when they’re open, and not letting up when things calm down.* [S11]

To ignite social transformation, speakers and participants offered bold visions to address persistent inequities in pandemic planning and recovery, and beyond:

- Recognize the interconnectedness of all planetary elements
- Invest in the ecological and social determinants of health for all communities in government policies
- Apply intersectoral policy approaches resourced by wellbeing budgets
- Transform health and social systems to better account for equity for example, collect race and equity data and implement appropriate programs and services
- Develop alternative social, economic and political systems and approaches grounded in an ethic of care, compassion, trust-building and togetherness

*So when you start to look at pandemics like COVID-19, we start to understand that it’s not just human beings in this world, that the plants, the animals, the birds, the fish, the land, the soil, the water, it’s all connected so we start to heal Mother Earth, we can start to heal people as well*. [S1]

## Discussion

COVID-19 was declared a global pandemic by the World Health Organization on 13 March 2020. This declaration was rapidly followed by the implementation of public health measures by different levels of governments across the world. In Canada, lockdown and physical distancing measures were implemented by federal, provincial/territorial and regional governments. Our findings support that COVID-19 and the subsequent public health responses had unique and deep impacts on health that followed lines of existing structural inequities.

Despite past recommendations on integrating equity and health promotion principles into pandemic planning and preparedness (17–19) and notwithstanding warnings of future pandemics resulting from significant ecological changes (18,27), our findings support that governments and health and social systems did not adequately prepare and plan for equity. Further, the early responses to COVID-19 strongly suggest that governments were ill-prepared, and that health and social systems did not apply core health promotion principles and approaches in pandemic planning. Where equity was addressed, it appears to have been through later responses, as an afterthought rather than an initial driver or part of the emergency preparedness planning.

For equity-oriented researchers, practitioners and communities, the negative impacts did not come as a surprise. Instead they were experienced as a reverberation of generations of activism, advocacy, research and ongoing attempts to transform inequitable and oppressive social structures. Our findings emphasize that precarity was already built into the societal fabric. As a result, the existing social and health systems were destined to fail many parts of society, with communities at the margins bearing more than their fair share of the burdens brought on by COVID-19.

Community organizing and mobilization played a protective role. The inherent resilience of communities filled gaps left by frail and toxic health and social systems which were slow or unresponsive. The capacity of communities to mobilize, however necessary, does not meet the need for widespread social support and protection, nor does it absolve governments from their responsibility to ensure the health and wellbeing of all people.

Equity and social justice are manifestations of an ethic of care (28) and communitarian perspectives (29). If ethical principles and values are to be applied to pandemic preparedness and responses, we need to be attentive to questions of implementation and structural inequities (13). This will require a stronger engagement between health promotion scholars and practitioners, and infectious diseases and emergency preparedness specialists (30).

Speakers and participants consistently expressed a strong desire to move toward a more just and equitable society, going ‘back to better’ rather than ‘back to normal’ in a post-COVID-19 world. Stronger critiques of public health practice and public policy that draw on critical health promotion principles are needed to inform pandemic responses, so they reflect a better normal. Critiques should offer up bold visions of how to reorganize society for better health and wellbeing. Health promotion principles coupled with other approaches to social, political and economic change offer a path forward. This raises the questions: Are the current mobilizations and policy innovations expressions of a moment, or can they serve as the basis for social transformation, growing into a sustained movement for health and health equity? Can increased public consciousness be sustained beyond the pandemic and transformed into political action to improve health equity?

Social movement (31–34) and systems theories (35–37) offer vital insights on how health promotion research, practice and policy can contribute to current momentum to move toward a better normal. Systems theory tells us that systems are deeply connected and offer different points of leverage for action (35). Social movement theory explicitly accounts for conflict and calls for mobilizing structures, resonant frames and political opportunities. COVID-19 has opened up political opportunities which, if fully exploited, can lead to significant social transformation for health beyond pandemic planning and responses. Together, both theoretical approaches speak to the pertinence of narrative practices that shift the fundamental assumptions that underpin systems. These narratives, coupled with new and existing mobilizing structures, can be directed to planetary health disruptions and building health-promoting socioeconomic and political systems (5,38). Concretely this means:

- Apply a ‘whole community’ approach that engages individuals and organizations in public health emergency planning to strengthen community capacity during response and recovery (39) and allow for inclusion of community-based risks and lived experiences (40).
- Strategize with non-health sector partners to design a just and sustainable future.
- Act with non-health sector partners to disrupt oppressive systems and invest in healthy communities.
- Engage in effective message framing and media advocacy to maintain these equity issues, and their solutions, in the public consciousness.

## References

[1] Greenwood M de Leeuw S Lindsay NM (eds). Determinants of Indigenous Peoples’ Health in Canada: Beyond the Social. Toronto: Canadian Scholars’ Press; 2015.

[2] Paradies Y Ben J Denson N Elias A Priest N Pieterse A , et al. Racism as a determinant of health: a systematic review and meta-analysis. PLoS One. 2015; 10: e0138511. 26398658 10.1371/journal.pone.0138511 PMC4580597

[3] Navarro V Shi L. The political context of social inequalities and health. Soc Sci Med. 2001; 52: 481–491. 11330781 10.1016/s0277-9536(00)00197-0

[4] Pan American Health Organization. Just Societies: Health Equity and Dignified Lives. Executive Summary of the Report of the Commission of the Pan American Health Organization on Equity and Health Inequalities in the Americas [Internet]. Revised ed. Washington, DC: Pan American Health Organization; 2019 [cited 2020 July 13]. Available from: https://iris.paho.org/handle/10665.2/51570

[5] Raphael D. Bryant T. Mikkonen J. Raphael A. (2020). Social determinants of health: The Canadian facts [Internet]. Oshawa, ON: Ontario Tech University Faculty of Health Sciences and Toronto, ON: York University School of Health Policy and Management. Available from: http://www.thecanadianfacts.org/

[6] Rutter PD Mytton OT Mak M Donaldson LJ. Socio-economic disparities in mortality due to pandemic influenza in England. Int J Public Health. 2012; 57: 745–750. 22297400 10.1007/s00038-012-0337-1

[7] National Collaborating Centre for Aboriginal Health. The 2009 H1N1 Influenza Pandemic Among First Nations, Inuit and Métis Peoples in Canada: Epidemiology and Gaps in Knowledge. Prince George, BC: NCCAH; 2016.

[8] Centers for Disease Control and Prevention. 2009 pandemic influenza A (H1N1) virus infections—Chicago, Illinois, April–July 2009. MMWR Morb Mortal Wkly Rep. 2009; 58: 913–918. 19713879

[9] Centers for Disease Control and Prevention. Deaths related to 2009 pandemic influenza A (H1N1) among American Indian/Alaska natives—12 states, 2009. MMWR Morb Mortal Wkly Rep. 2009; 58: 1341–1344. 20010508

[10] Klein SL Passaretti C Anker M Olukoya P Pekosz A. The impact of sex, gender and pregnancy on 2009 H1N1 disease. Biol Sex Differ. 2010; 1: 5. 21208468 10.1186/2042-6410-1-5 PMC3010100

[11] Campbell VA Gilyard JA Sinclair L Sternberg T Kailes JI. Preparing for and responding to pandemic influenza: implications for people with disabilities. Am J Public Health. 2009; 99: S294–S300. 19797741 10.2105/AJPH.2009.162677 PMC4504380

[12] World Health Organization; Health and Welfare Canada; Canadian Public Health Association. Ottawa: Charter for Health Promotion [Internet]. 1986 [cited 2020 July 13]. Available from: https://www.canada.ca/content/dam/phac-aspc/documents/services/health-promotion/population-health/ottawa-charter-health-promotion-international-conference-on-health-promotion/charter.pdf/

[13] DeBruin D Liaschenko J Marshall MF. Social justice in pandemic preparedness. Am J Public Health. 2012; 102: 586–591. 22397337 10.2105/AJPH.2011.300483 PMC3489368

[14] Kayman H Ablorh-Odjidja A. Revisiting public health preparedness: incorporating social justice principles into pandemic preparedness planning for influenza. J Public Health Manag Pract. 2006; 12: 373–380. 16775535 10.1097/00124784-200607000-00011

[15] Plough A Bristow B Fielding J Caldwell S Khan S. Pandemics and health equity: lessons learned from the H1N1 response in Los Angeles County. J Public Health Manag Pract. 2011; 17: 20–27. 21135657 10.1097/PHH.0b013e3181ff2ad7

[16] National Advisory Committee on SARS and Public Health. Learning from SARS: Renewal of Public Health in Canada. Ottawa: Health Canada; 2003.

[17] Khan Y Fazli G Henry B de Villa E Tsamis C Grant M , et al. The evidence base of primary research in public health emergency preparedness: a scoping review and stakeholder consultation. BMC Public Health. 2015; 15: 432. 25925775 10.1186/s12889-015-1750-1 PMC4415223

[18] Stephen C. Rethinking pandemic preparedness in the Anthropocene. Healthc Manage Forum. 2020; 33: 153–157. 31698955 10.1177/0840470419867347

[19] Tam T. Fifteen years post-SARS: key milestones in Canada’s public health emergency response. Can Commun Dis Rep. 2018; 44: 98-101. 31007618 10.14745/ccdr.v44i05a01 PMC6449094

[20] Canadian Pandemic Influenza Preparedness: Planning Guidance for the Health Sector [Internet]. Ottawa, ON: Government of Canada; 2018 [cited 2020 July 13]. Available from: https://www.canada.ca/en/public-health/services/flu-influenza/canadian-pandemic-influenza-preparedness-planning-guidance-health-sector.html 10.14745/ccdr.v44i01a02 PMC5937063 29770091

[21] Public Health Agency of Canada. Vulnerable Populations and COVID-19 [Internet]. Ottawa, ON: Public Health Agency of Canada; 2020 May 28 [cited 2020 July 13]. Available from: https://www.canada.ca/en/public-health/services/publications/diseases-conditions/vulnerable-populations-covid-19.html/

[22] National Collaborating Centre for Determinants of Health. Conversation Series: Health Equity, Determinants of Health and COVID-19 [webcast]. Antigonish, NS: National Collaborating Centre for Determinants of Health; 2020 [cited 2020 July 13]. 5 videos. Available from: https://nccdh.ca/index.php?/workshops-events/entry/COVID-19-webinar-conversation-series

[23] Braun V Clarke V. Using thematic analysis in psychology. Qual Res Psychol. 2006; 3: 77–101.

[24] Nowell LS Norris JM White DE Moules NJ. Thematic analysis: striving to meet the trustworthiness criteria. Int J Qual Methods. 2017; 16: 1609406917733847.

[25] Stevens G O’Donnell VL Williams L. Public domain or private data? Developing an ethical approach to social media research in an inter-disciplinary project. Educ Res Eval. 2015; 21: 154–167.

[26] Government of Canada [Internet]. Canada Emergency Response Benefit (CERB). Ottawa, ON: Government of Canada; 2020 [cited 2020 July 13]. Available from: https://www.canada.ca/en/services/benefits/ei/cerb-application.html/

[27] Canadian Public Health Association. Global Change and Public Health: Addressing the Ecological Determinants of Health [Internet]. Ottawa, ON: Canadian Public Health Association; 2015 [cited 2020 Jan 12]. Available from: https://www.cpha.ca/sites/default/files/assets/policy/edh-discussion_e.pdf/

[28] Falk-Rafael A Betker C. Witnessing social injustice downstream and advocating for health equity upstream: “the trombone slide” of nursing. ANS Adv Nurs Sci. 2012; 35: 98–112. 22429948 10.1097/ANS.0b013e31824fe70f

[29] MacDonald M . Introduction to Public Health Ethics 3: Frameworks for Public Health Ethics. Montréal, QC: National Collaborating Centre for Healthy Public Policy; 2015.

[30] Van den Broucke S . Why health promotion matters to the COVID-19 pandemic, and vice versa. Health Promot Int. 2020; 35: 181–186. 32297931 10.1093/heapro/daaa042 PMC7184433

[31] Brown P Zavestoski S. Social movements in health: an introduction. Sociol Health Illn. 2004; 26: 679–694. 15383036 10.1111/j.0141-9889.2004.00413.x

[32] Morris AD Mueller CM. Frontiers in Social Movement Theory. New Haven, CT: Yale University Press; 1992.

[33] Touraine A. An introduction to the study of social movements. Soc Res. 1985; 52: 749–787.

[34] Roundtable on Population Health Improvement; Roundtable on the Promotion of Health Equity and the Elimination of Health Disparities; Board on Population Health and Public Health Practice; Institute of Medicine. Supporting a Movement for Health and Health Equity: Lessons from Social Movements: Workshop Summary. Washington, DC: National Academies Press; 2014. 25590123

[35] Carey G Crammond B. Systems change for the social determinants of health. BMC Public Health. 2015; 15: 1–10. 26168785 10.1186/s12889-015-1979-8 PMC4501117

[36] Pauly B Shahram SZ van Roode T Strosher HW MacDonald M. Reorienting Health Systems Towards Health Equity: The Systems Health Equity Lens (SHEL). Victoria, BC: The Equity Lens in Public Health (ELPH) Research Project; 2018.

[37] Xia S Zhou X Liu J. Systems thinking in combating infectious diseases. Infect Dis Poverty. 2017; 6: 1–7. 28893320 10.1186/s40249-017-0339-6 PMC5594605

[38] National Collaborating Centre for Determinants of Health. Common Agenda for Public Health Action on Health Equity. Antigonish, NS: National Collaborating Centre for Determinants of Health; 2016.

[39] Ramsbottom A O’Brien E Ciotti L Takacs J. Enablers and barriers to community engagement in public health emergency preparedness: a literature review. J Community Health. 2018; 43: 412–420. 28840421 10.1007/s10900-017-0415-7 PMC5830497

[40] Khan Y O’Sullivan T Brown A Tracey S Gibson J Généreux M , et al. Public health emergency preparedness: a framework to promote resilience. BMC Public Health. 2018; 18: 1344. 10.1186/s12889-018-6250-7 PMC6280369 30518348

